# Synthesis of Fullerenes from a Nonaromatic Chloroform through a Newly Developed Ultrahigh-Temperature Flash Vacuum Pyrolysis Apparatus

**DOI:** 10.3390/nano11113033

**Published:** 2021-11-12

**Authors:** Hong-Gang Zhang, Ya-Qi Zhuo, Xiao-Min Zhang, Leng Zhang, Piao-Yang Xu, Han-Rui Tian, Shui-Chao Lin, Qianyan Zhang, Su-Yuan Xie, Lan-Sun Zheng

**Affiliations:** State Key Laboratory for Physical Chemistry of Solid Surfaces, iChEM (Collaborative Innovation Center of Chemistry for Energy Materials), Department of Chemistry, Xiamen University, Xiamen 361005, China; bingfengdmy@163.com (H.-G.Z.); zyq139795@163.com (Y.-Q.Z.); zxmcomet@163.com (X.-M.Z.); lengzhang0923@163.com (L.Z.); 20170155073@xmu.edu.cn (P.-Y.X.); tianhanrui@xmu.edu.cn (H.-R.T.); syxie@xmu.edu.cn (S.-Y.X.); lszheng@xmu.edu.cn (L.-S.Z.)

**Keywords:** fullerenes, flash vacuum pyrolysis, nanocarbon, pyrolysis apparatus

## Abstract

The flash vacuum pyrolysis (FVP) technique is useful for preparing curved polycyclic aromatic compounds (PAHs) and caged nanocarbon molecules, such as the well-known corannulene and fullerene C_60_. However, the operating temperature of the traditional FVP apparatus is limited to ~1250 °C, which is not sufficient to overcome the high energy barriers of some reactions. Herein, we report an ultrahigh-temperature FVP (UT-FVP) apparatus with a controllable operating temperature of up to 2500 °C to synthesize fullerene C_60_ from a nonaromatic single carbon reactant, i.e., chloroform, at 1350 °C or above. Fullerene C_60_ cannot be obtained from CHCl_3_ using the traditional FVP apparatus because of the limitation of the reaction temperature. The significant improvements in the UT-FVP apparatus, compared to the traditional FVP apparatus, were the replacement of the quartz tube with a graphite tube and the direct heating of the graphite tube by impedance heating instead of indirect heating of the quartz tube using an electric furnace. Because of the higher temperature range, UT-FVP can not only synthesize fullerene C_60_ from single carbon nonaromatic reactants but sublimate some high-molecular-weight compounds to synthesize larger curved PAHs in the future.

## 1. Introduction

During the past three decades, fullerenes, carbon nanotubes, and graphene/graphene quantum dots have achieved great progress as representative carbon nanomaterials [1,2,3,4,5,6,7,8]. Fullerene C_60_, one of the allotropes of carbon, was synthesized for the first time in 1985 by laser evaporation of graphite [1]. These types of fullerene carbon nanomaterials with high conjugated cage-like structures have attracted significant attention owing to their special properties [9,10] and applications in areas such as biomedicine, catalysis, superconduction, and nonlinear optics [11,12,13,14,15], and especially in the electronic applications of photovoltaics, organic thermoelectrics, and electrochemical transistors [16,17,18,19,20]. These important applications make scientists have a higher demand for greater amounts and new structures of fullerenes, which require a clear understanding of the fullerene formation mechanism. However, the yield of fullerene produced using laser evaporation of graphite is very low, which is not conducive to intensive studies of fullerene. To improve the yield of fullerene, several different preparation methods have been developed to realize the macrosynthesis of fullerenes, including microwave plasma, glow discharge, arc discharge, and flame combustion [21,22,23,24,25,26]. The common feature of these physicochemical synthesis methods is that the experimental temperature reaches thousands of degrees, and the reaction temperature cannot be controlled. Under such high-energy reaction conditions of thousands of degrees, stable carbon clusters such as fullerenes C_60_ and C_70_ can be produced. However, some of the relatively unstable or metastable carbon clusters, such as smaller fullerenes or some intermediate products that form fullerenes or other important carbon clusters, are difficult to sustain. To some extent, the absence of these metastable carbon clusters creates severe constraints for experimental investigations of fullerene and other carbon cluster formations [27,28,29,30,31,32,33,34]. Therefore, preparation methods with relatively milder reaction conditions and relatively controllable temperature are more likely to yield metastable precursors of fullerenes or intermediate products, which can then aid in further exploring the formation mechanism of fullerenes.

In addition to techniques such as microwave plasma, glow discharge, arc discharge, and flame combustion [21,22,23,24,25,26], flash vacuum pyrolysis (FVP) has been used to synthesize fullerenes. It generates products by transient thermal excitation of gas-phase reactants in vacuum followed by rapid cooling of products [35,36,37,38,39,40]. In 1993, for the first time, Taylor and Kroto et al., synthesized fullerenes C_60_ and C_70_ using the pyrolysis method by pyrolyzing naphthalene [38]. Additionally, studies reported the use of PAHs, such as corannulene and benzo[k]fluoranthene, as precursors for the synthesis of fullerenes using the pyrolysis technique [39]. In 2002, Scott et al., used the FVP technique to realize the rational chemical synthesis of fullerene C_60_ from a predesigned precursor of a chlorinated PAH molecule [40]. All these pyrolysis reactions were conducted under mild conditions (650–1200 °C) in quartz tubes. The ability to control the reaction temperature is an important feature of FVP; this temperature can be adjusted using an electric furnace according to the requirements of the reaction. The operating temperature of the FVP apparatus is usually not higher than 1250 °C, which is mainly limited by the characteristics of the heat units and quartz materials. However, when using the FVP apparatus, some reaction barriers are too high to overcome below 1250 °C. A FVP apparatus with a higher operating temperature is expected to provide sufficient energy for some specific reactions. Therefore, it is crucial to extend the operating temperature limit of the existing FVP apparatus.

In the FVP technique, aromatic compounds, such as corannulene [39], benzo[k]fluoranthene [39], or naphthalene [38] are usually used as the starting materials for the synthesis of fullerenes. The synthesis of fullerenes from nonaromatic compounds using the FVP technique has not been reported yet. Generally, it is believed that fullerenes can be synthesized from nonaromatic compounds only through common physicochemical synthesis methods with high energy. Additionally, according to our previous research, a series of fullerenes and their derivatives were synthesized from chloroform and carbon tetrachloride using common physicochemical synthesis methods with high energy, such as arc discharge, glow discharge, and microwave plasma [21,22,41,42]. 

Here, we developed a new ultrahigh-temperature FVP (UT-FVP) apparatus, with a controllable operating temperature higher than that of traditional FVP but lower than that of common physicochemical synthesis methods, with more suitable high energy for the pyrolysis reaction. The proposed FVP apparatus was employed for the novel synthesis of fullerenes from a nonaromatic single carbon reactant, chloroform, under relatively mild reaction conditions. To increase the operating temperature of UT-FVP, the traditional quartz tube, which is typically heated indirectly by an electric furnace, was replaced with a graphite tube, which was heated directly by the electrode. To the best of our knowledge, this is the first study to report the synthesis of fullerenes from nonaromatic chloroform using the FVP technique.

## 2. Materials and Methods

**Figure 1:**
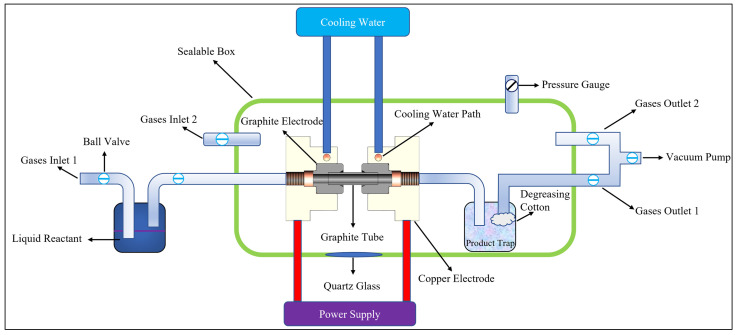
Schematic of the newly developed ultrahigh-temperature flash vacuum pyrolysis (UT-FVP) apparatus.

As shown in Figure 1 and Table S1, the proposed UT-FVP apparatus consisted of six units: the gas inlet/outlet, electrode heating control, cooling electrode, graphite tube, product trap, and vacuum unit. Gas inlet/outlet 1 and the vacuum unit were responsible for adjusting the vacuum environment required for the pyrolysis reactions and for transporting the gaseous reactants or volatile liquid reactants to the high-temperature reaction zone (graphite tube lumen); this was followed by the rapid transportation of the generated gaseous products to the product trap unit. The graphite tube and the electrode heating control were the core components of the UT-FVP apparatus. To prevent the copper electrodes from melting at high temperature, a transitional graphite electrode was embedded in the copper electrode, which circulated water to cool the copper electrode. The graphite tube was heated by impedance heating, and the temperature of the graphite tube was regulated by adjusting the voltage applied to it. Generally, the connections among the gas inlet, graphite tube, and product trap unit were not completely sealed. Therefore, the graphite tube, product trap unit, and connection between the gas inlet and the graphite tube were integrated into a sealed stainless-steel box to offer a low-pressure reaction environment in an inert atmosphere. A quartz glass observation window was arranged on the wall of the sealed box, through which not only was the entire experimental phenomenon observed, but the pyrolysis reaction temperature was measured in real time using an infrared thermometer. Additionally, gas inlet/outlet 2 and the vacuum pump were designed on the box to quickly inflate and create vacuum in the box (Figure S1). 

Considering that the melting point of graphite (more than 3000 °C) is much higher than that of quartz, a graphite tube was used to replace the quartz tube in the traditional UT-FVP apparatus. Additionally, the indirect electric furnace heating method in the traditional FVP device was replaced by a direct electrode heating the graphite tube in the UT-FVP apparatus. Because of the significant changes in these two aspects, the operating temperature range of the new UT-FVP apparatus increased from 300–1250 °C for the traditional FVP device to 300–2500 °C. The UT-FVP apparatus provided faster heating by directly heating the graphite tube with the electrode. In the traditional FVP apparatus, it usually takes 1–2 h for the quartz tube to heat up to 1200 °C. However, the graphite tube in the UT-FVP apparatus took only 10 s to heat up to 2500 °C (Figure S2). The graphite tube acted as a heating body and a reaction chamber simultaneously; therefore, compared with the traditional FVP device, the UT-FVP device possessed several advantages, such as simpler operation, higher working efficiency, and higher operating temperature.

We conducted the pyrolysis of nonaromatic CHCl_3_ using the UT-FVP apparatus, which required a common physicochemical synthesis method with high energy compared with the relatively milder FVP approach. First, the reaction chamber was adjusted to a low-pressure helium environment of 90 Torr; then, the stainless-steel box was sealed, vacuum was created through gas outlet 2 until the pressure reached a value less than 10^−3^ Torr, and finally, helium was injected through gas inlet 2 until the pressure reached ~90 Torr. Subsequently, the graphite tube was heated to a preset temperature by adjusting the voltage of the copper electrodes, followed by bubbling CHCl_3_ vapor with helium and rapidly carrying it through the high-temperature zone of the hot graphite tube via gas inlet 1; the vacuum pressure of the graphite tube was maintained at a dynamic equilibrium of ~100 Torr during the pyrolysis reaction, and the reaction lasted for approximately 2 min. Finally, the carbon soot products were collected in the trap.

## 3. Results and Discussion

**Figure 2:**
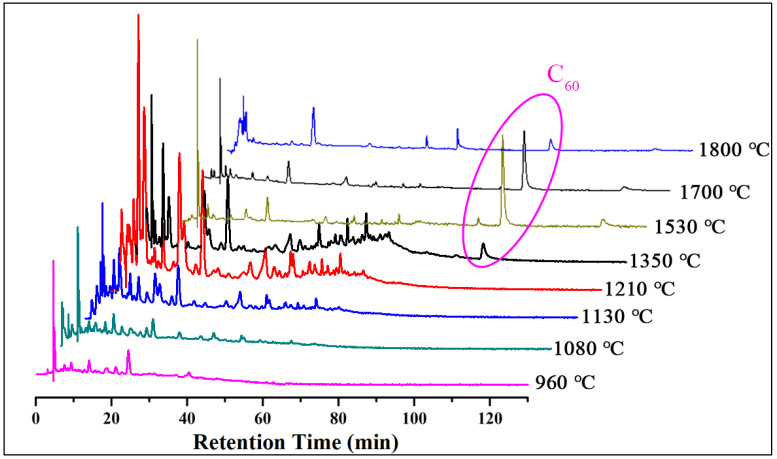
HPLC-UV (330 nm) chromatogram of the product from the pyrolysis of CHCl_3_ at the temperature range of ~960–1800 °C.

**Figure 3:**
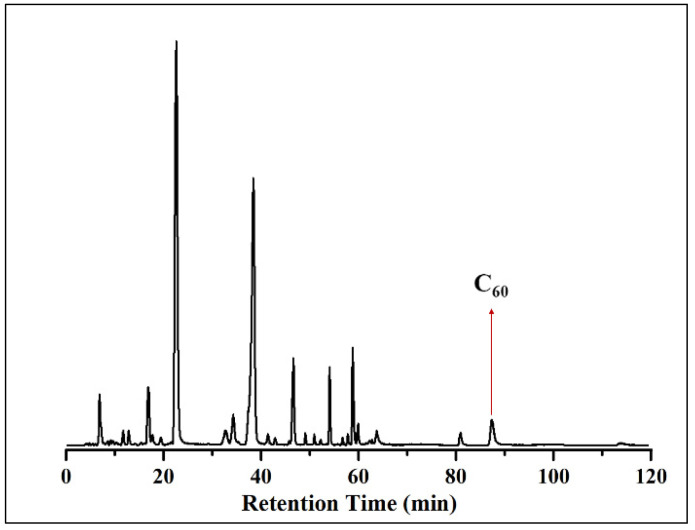
HPLC-MS chromatogram of the soot products from the pyrolysis of CHCl_3_ at 1530 °C.

The pyrolysis temperature plays a crucial role in UT-FVP because it provides energy for the pyrolysis reaction and determines the products that can be obtained. Herein, several temperature control experiments were conducted in the temperature range of 960–1800 °C (Figure 2) while keeping the other experimental parameters unchanged. According to the high-performance liquid chromatography with UV detection coupled with mass spectrometry (HPLC-UV-MS) results (Table S2), the signal of fullerene C_60_ was first observed with a retention time of 82–88 min, when the pyrolysis temperature increased to 1350 °C. The yield of C_60_ increased with increasing temperature, reaching a maximum at ~1530 °C, after which the yield decreased with further increase in temperature. Noticeably, there was no signal peak of C_60_ in the HPLC-UV chromatogram (Table S3) in the temperature range of 960–1210 °C, indicating that no C_60_ was obtained. The results agreed well with the experimental results obtained using the traditional FVP device, i.e., when chloroform was pyrolyzed at 1200 °C using the traditional FVP apparatus (Figure S3). The temperature control experiment results suggest that the optimum temperature for the pyrolysis of CHCl_3_ is ~1530 °C (Figure 3).

In most fullerene synthesis techniques, such as laser evaporation and arc discharge, helium plays a crucial role in the formation of fullerenes from pure graphite [1,13]. The low-cost inert gas, i.e., nitrogen, cannot be used as a carrier gas because it decreases the yield of fullerene C_60_ and C_70_ [43]. In addition to helium gas, nitrogen gas was selected as a carrier gas in our UT-FVP apparatus. The HPLC-UV-MS results showed that there was no difference in the yield of fullerenes when helium and nitrogen were used as carriers (Figure S4). This indicated that fullerene C_60_ could also be prepared from CHCl_3_ in nitrogen under an inert atmosphere. The optimal pressures of helium and nitrogen were determined as 100 and 150 Torr, respectively (Figure S5), which were consistent with the previously reported optimal pressures for fullerene formation [23]. 

Based on the standard curve diagram drawn according to peak areas of the HPLC spectrum to concentrations of C_60_, we determined that the mass fraction of C_60_ in soot was ~1.44% (Figure S6). Although the yield of C_60_ was not outstanding compared with those of other methods, the relative yield of C_60_ can be improved, and various parameters of the device are being optimized. In addition to fullerenes, the soot products contained a large number of chlorinated carbon clusters. The two isotopes of chlorine exhibited a special abundance distribution (^35^Cl/^37^Cl = 75.77:24.23); therefore, the numbers of chlorine atoms in these chlorinated carbon clusters were clearly determined based on the distribution of chlorine isotope peaks in compounds displayed by the HPLC-MS chromatogram (Figure S7). On comparing the simulated isotopic distribution with the isotopic distribution recorded by MS, it was easy to determine the molecular formula from the number of chlorine atoms and carbon atoms in each chlorinated carbon cluster. The analysis of the molecular formula of chlorinated carbon clusters showed that the pyrolysis products contained a large number of polychlorinated, polycyclic aromatic hydrocarbons, and these chlorinated carbon clusters (C_8_Cl_6_, C_12_Cl_8_, C_14_Cl_8_, C_18_Cl_10_, and C_20_Cl_12_) were the possible intermediates or precursors for the formation of fullerenes. Compared with the experimental results (CHCl_3_ as the reactant) based on the microwave synthesis of fullerenes [21], more small chlorinated carbon clusters, hydrochlorinated carbon clusters and fullerene derivatives, such as C_14_Cl_10_, C_16_Cl_12_, C_20_Cl_12_, C_10_H_4_Cl_6_, C_20_H_8_Cl_8_, C_62_H_8_, C_70_H_4_, etc., were observed during the pyrolysis of CHCl_3_ to fullerene, which indicated that more intermediate carbon clusters for the formation of fullerenes could be obtained by this new pyrolysis technique under relatively milder reaction conditions. We tried to determine the specific structure of these chlorinated carbon clusters and provided more information on the formation of fullerenes in the follow-up work.

**Figure 4:**
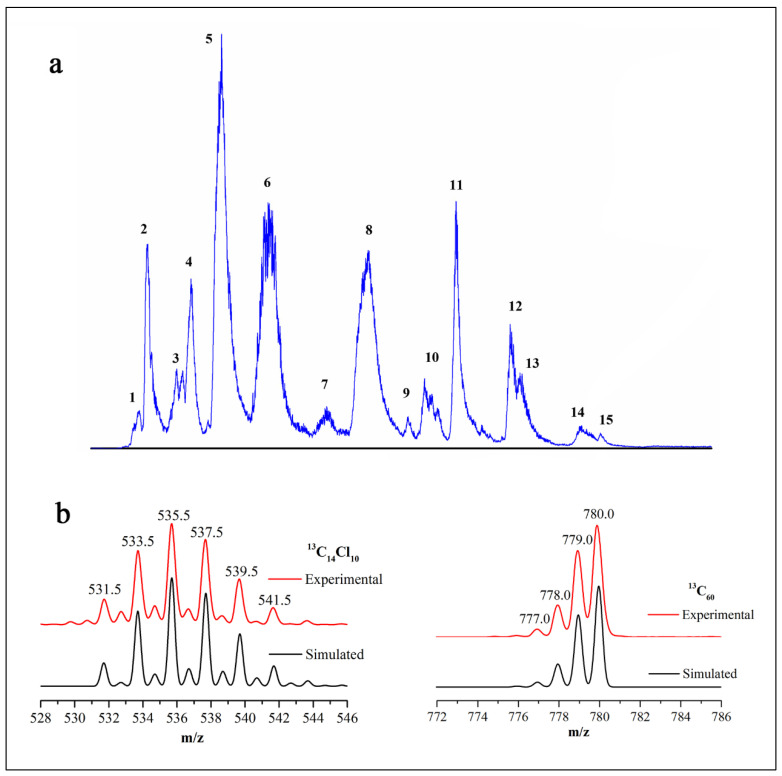
(**a**) HPLC-MS chromatogram of soot products from the pyrolysis of ^13^CHCl_3_ at 1740 °C. Peaks are labeled by numbers, and their molecular formulae are suggested from their isotope distributions in mass spectra: (1) ^13^C_10_Cl_8_; (2) ^13^C_6_Cl_5_O; (3) ^13^C_12_Cl_9_O; (4) ^13^C_12_Cl_8_; (5) ^13^C_10_Cl_7_O; (6) ^13^C_14_Cl_10_; (7) impurity; (8) ^13^C_12_Cl_8_; (9) ^13^C_14_Cl_9_O; (10) ^13^C_18_Cl_12_; (11) ^13^C_16_Cl_10_; (12) ^13^C_18_Cl_10_; (13) ^13^C_22_Cl_12_; (14) ^13^C_24_Cl_12_; and (15) ^13^C_60._ (**b**) The experimental and theoretically simulated mass spectra of ^13^C_14_Cl_10_ and ^13^C_60_ (^13^C = 99%).

As described above, the pyrolytic reaction occurred in a high-temperature graphite tube composed of layered aromatic carbons. To determine whether all the carbons in the pyrolytic products came from CHCl_3_ or part of the graphite tube, we chose ^13^C-labeled CHCl_3_ (purity: 99%) as the reactant and performed the pyrolysis experiment at 1740 °C. The results of HPLC-MS showed that the product of C_60_ was ^13^C_60_ and that the chlorinated carbon cluster was ^13^C_14_Cl_10_. The isotopic peak abundance of ^13^C_60_ was in good agreement with the theoretical simulation, as shown in Figure 4, which revealed that all the carbon atoms of fullerene C_60_ and chlorinated carbon clusters came from the reactant ^13^CHCl_3_. The isotope (^13^C) experiments ruled out the possibility that the graphite tube participated in the reaction. Additionally, when no reactants were introduced, no fullerene C_60_ was produced even when the graphite tube was heated to 2500 °C, but the tube gradually became thinner and eventually fused. On increasing the temperature, the fullerene C_60_ product reappeared, which was similar to the synthesis of fullerene by evaporation of graphite (Figure S8).

## 4. Conclusions

In summary, we developed a new UT-FVP apparatus and synthesized fullerenes from CHCl_3_ under relatively mild and controllable reaction conditions for the first time. Compared with that of the traditional FVP apparatus, the operating temperature of the UT-FVP device increased, which can be attributed to the replacement of the quartz tube with a graphite tube and the use of an electrode to directly heat the graphite tube. Theoretically, the working temperature of UT-FVP reached approximately 3000 °C, while experimentally, it was close to 2500 °C. In UT-FVP, the pyrolysis results of CHCl_3_ suggested that a high operating temperature provided sufficient energy to overcome the high reaction barrier. The pyrolysis results of ^13^C-labeled CHCl_3_ at 1740 °C suggested that all the carbon in the pyrolysis products came from the reactant ^13^CHCl_3_, which ruled out the possibility of the graphite tube participating in the reaction. Compared with arc discharge and other common high-energy synthesis technologies, UT-FVP technology not only reduced the production cost by using nonaromatic compounds and nitrogen as raw materials and carrier gas, respectively, but provided relatively more controllable pyrolytic temperature and milder pyrolytic conditions, which might be helpful for further intensive studies on fullerene formation mechanisms. Additionally, in the future, this technology may be applied to synthesize curved PAHs with larger molecular weights.

## Data Availability

The data presented in this study are available on a reasonable request from the corresponding author.

## Supplementary Materials

The following are available online at https://www.mdpi.com/article/10.3390/nano11113033/s1, Figure S1: The graphic model of the sealable box in UT-FVP apparatus; Table S1: The main parameters of the UT-FVP apparatus; Table S2: The parameters of the mass spectrum; Table S3: The parameters of HPLC; Figure S2: The heating rate of the UT-FVP apparatus; Figure S3: The HPLC-MS chromatogram of the product from the pyrolysis of CHCl_3_ at 1100 °C and 1200 °C using traditional FVP apparatus; Figure S4: The HPLC-MS chromatogram of the product from the pyrolysis of CHCl_3_ at 1530 °C using inert gas N_2_ (red line) and He (blue line), respectively; Figure S5: Variation of C_60_ product concentration with vacuum pressure of reaction system. The red line means that the inert gas (N_2_ was used in the pyrolysis reaction, and the blue line means that the inert gas He was used in the pyrolysis reaction; Figure S6: The standard curve (red) of the UV (330 nm) chromatogram of C_60_ and the experimental data of CHCl_3_ (blue); Figure S7: Experimental and theoretically simulated mass spectra of the soot products; Figure S8: The HPLC-MS chromatograms of the soot products from the pyrolysis of the graphite tube at variable pyrolysis temperatures from 2400 °C.
