# Supplementary material for: Synthesis of Fullerenes from a Nonaromatic Chloroform through a Newly Developed Ultrahigh-Temperature Flash Vacuum Pyrolysis Apparatus
Source: Nanomaterials (Basel). 2021 Nov 12;11(11):3033. doi: 10.3390/nano11113033 (PMC8618344; doi:10.3390/nano11113033)
Supplement: Supplementary file 1 [file nanomaterials-11-03033-s001.zip › nanomaterials-1442341-supplementary.pdf]

nanomaterials

Supplementary Materials

Synthesis of Fullerenes from a Nonaromatic Chloroform through a Newly Developed Ultrahigh-Temperature Flash Vacuum Pyrolysis Apparatus

Hong-Gang Zhang, Ya-Qi Zhuo, Xiao-Min Zhang, Leng Zhang, Piao-Yang Xu, Han-Rui Tian, Shui-Chao Lin *, Qianyan Zhang *, Su-Yuan Xie and Lan-Sun Zheng

State Key Laboratory for Physical Chemistry of Solid Surfaces, iChEM (Collaborative Innovation Center of Chemistry for Energy Materials), Department of Chemistry, Xiamen University, Xiamen 361005, China; bingfengdmy@163.com (H.-G.Z.); zyq139795@163.com (Y.-Q.Z.); zxmcomet@163.com (X.-M.Z.); lengzhang0923@163.com (L.Z.); 20170155073@xmu.edu.cn (P.-Y.X.); tianhanrui@xmu.edu.cn (H.-R.T.); syxie@xmu.edu.cn (S.-Y.X.); lszheng@xmu.edu.cn (L.-S.Z.)

* Correspondence: sclin@xmu.edu.cn (S.-C.L.); xmuzhangqy@xmu.edu.cn (Q.Z.)

S1. Materials and Apparatus

All reagents were purchased from commercial sources and used without further purification. The sealable box of UT-FVP apparatus is shown in Figure S1. The main parameters of UT-FVP apparatus are shown in Table S1.

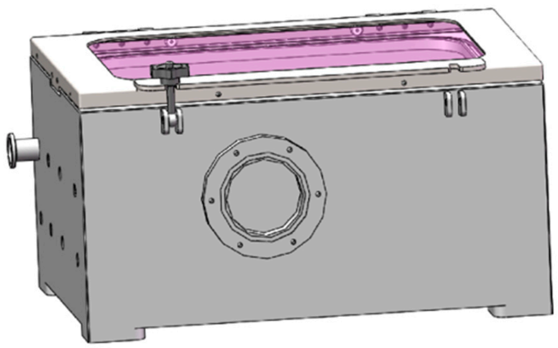

Figure S1. The graphic model of the sealable box in UT-FVP apparatus.

Table S1. The main parameters of UT-FVP apparatus.

Unit	Main parameters
Gas inlet/outlet	Flow rate: 0.4 L/min
	Temperature: room temperature
Electrode heating control	Current range: 0 ~ 500 A
	Temperature detection range: 0 ~2500 °C
Water cooling control	Flow rate: 5 L/min
	Temperature: room temperature
Graphite tube	Inner diameter: 6 mm; Outer diameter: 8 mm; Length: 33 mm.
Product trap	100 mL
Vacuum unit	Pumping rate: ~ 17 m^3/h<br>Pressure range: 10^{-3} ~ 10^5 pa

S2. Characterization

The soot was extracted with toluene using an ultrasound bath and then filtered using organic membrane at room temperature. The filtrate was concentrated and further separated and characterized by the high-performance liquid chromatography with UV detection coupled with mass spectrometry (HPLC-UV-MS) with the Agilent C18 column (4.6 × 250 mm, 5 μm particle size) coupled with the Agilent Time of Flight (TOF) 2000 mass spectrum, and the detail HPLC conditions are shown in Tables S2 and S3.

Since the peak area in the UV chromatogram is directly proportional to the content of fullerene C_{60}, the yield of fullerene can be determined according to the peak area under certain conditions. Specifically, the yield of fullerenes in soot products can be calculated by comparing the peak area of C_{60} in the sample with the peak area of C_{60} sample with standard curve (Figure S6). The standard curve of UV (330 nm) of fullerene C_{60} and HPLC data at 330 nm were analyzed by Shimadzu SPD-20A (Shimadzu CBM-20A, Shimadzu LC-6AD) with the Cosmosil 5PBB column (10 × 250 mm), and the mobile phase was toluene (chromatographic purity, flow rate: 4 ml/min).

Table S2. The parameters of Mass spectrum.

Unit	Main parameters
Ion Source	APCI
Ion Polarity	Negative
Nebulizer	45.0 psi
Dry Temperature	200 °C
Vaporizer Temperature	220 °C
Mass Range	50-2000 m/z

Table S3. The parameters of HPLC.

Time (min)	Methanol (%)	Ethanol (%)	Cyclohexane (%)	Velocity (ml/min)
0	100	0	0	0.8
15	85	15	0	0.8
30	85	15	0	0.8
60	55	10	35	0.8
130	55	10	35	0.8

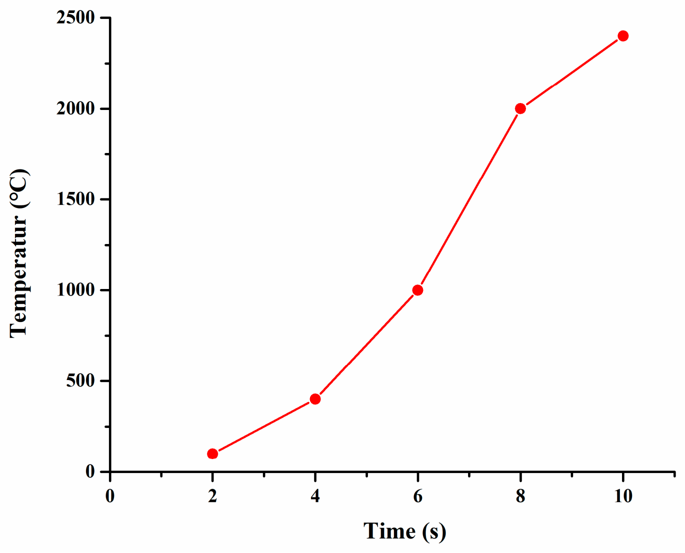

Figure S2. The heating rate of UT-FVP apparatus.

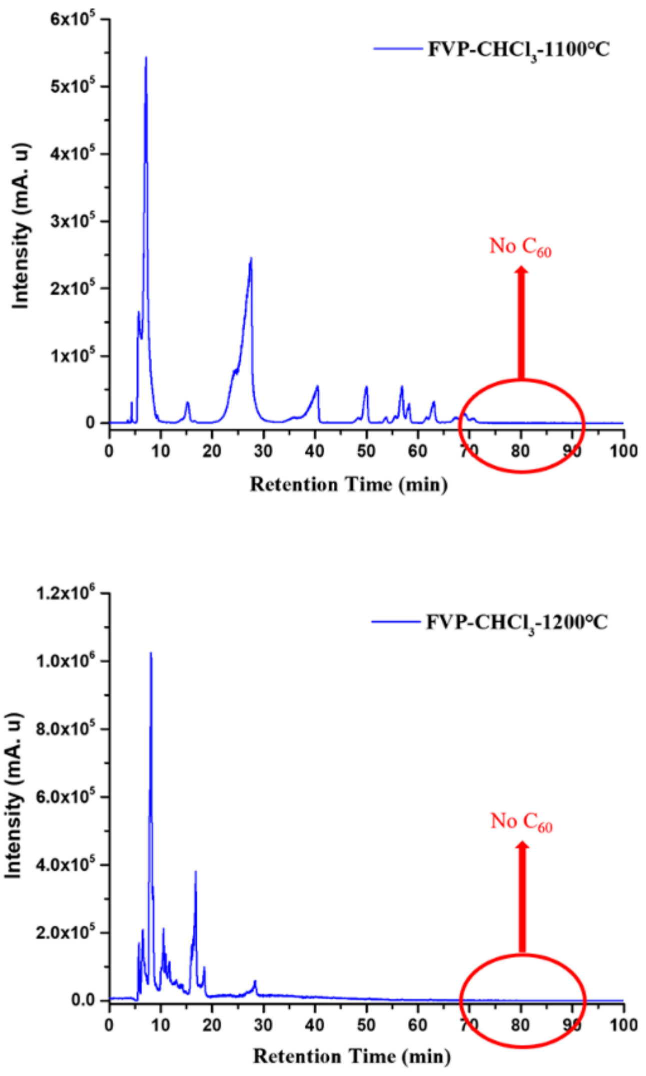

Figure S3. The HPLC-MS chromatogram of the product from the pyrolysis of $CHCl_3$ at 1100 °C and 1200 °C using traditional FVP apparatus.

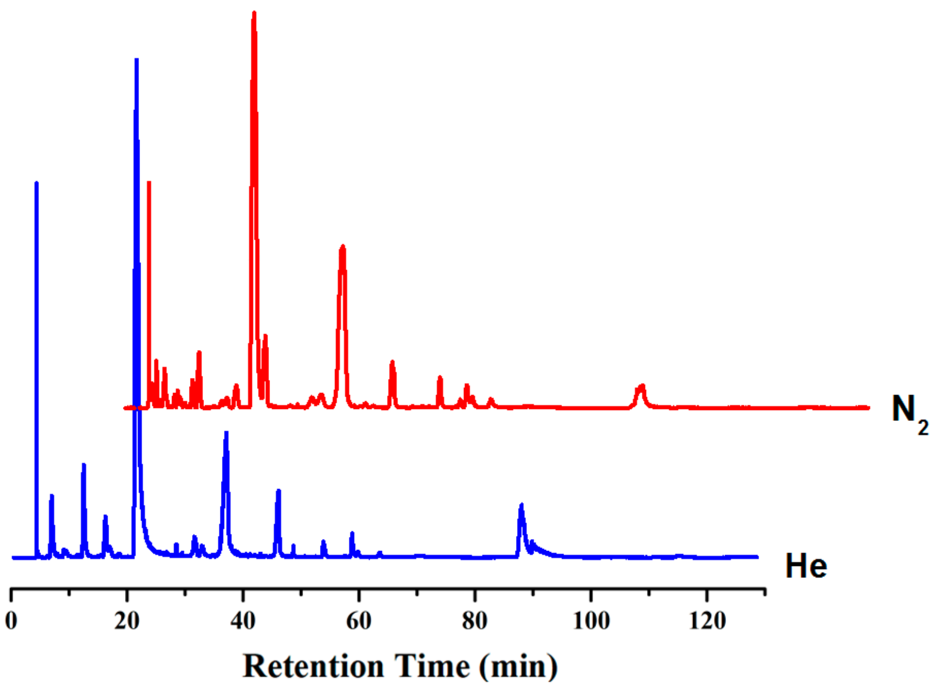

Figure S4. The HPLC-MS chromatogram of the soot product from the pyrolysis of $CHCl_3$ at 1530 °C using inert gas N_2 (red line) and He (blue line) respectively.

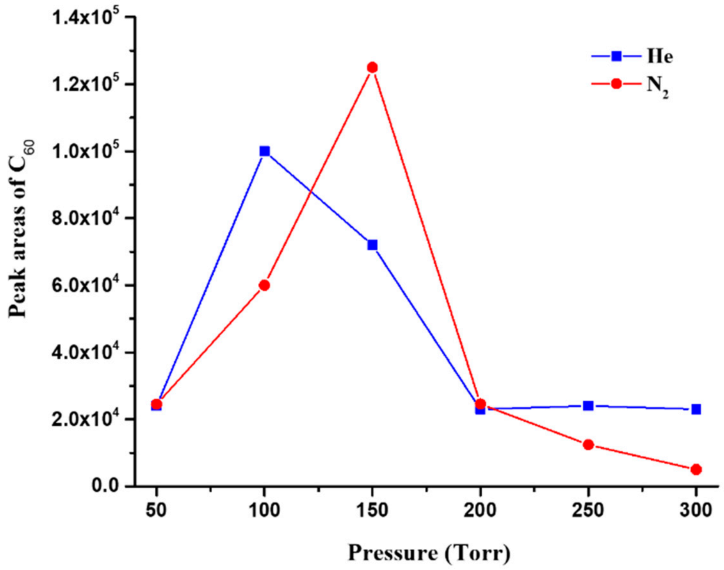

Figure S5. Variation of C_{60} product concentration with vacuum pressure of reaction system. The red line represents inert gas N_2 was used in the pyrolysis reaction, and the blue line means inert gas He was used in the pyrolysis reaction.

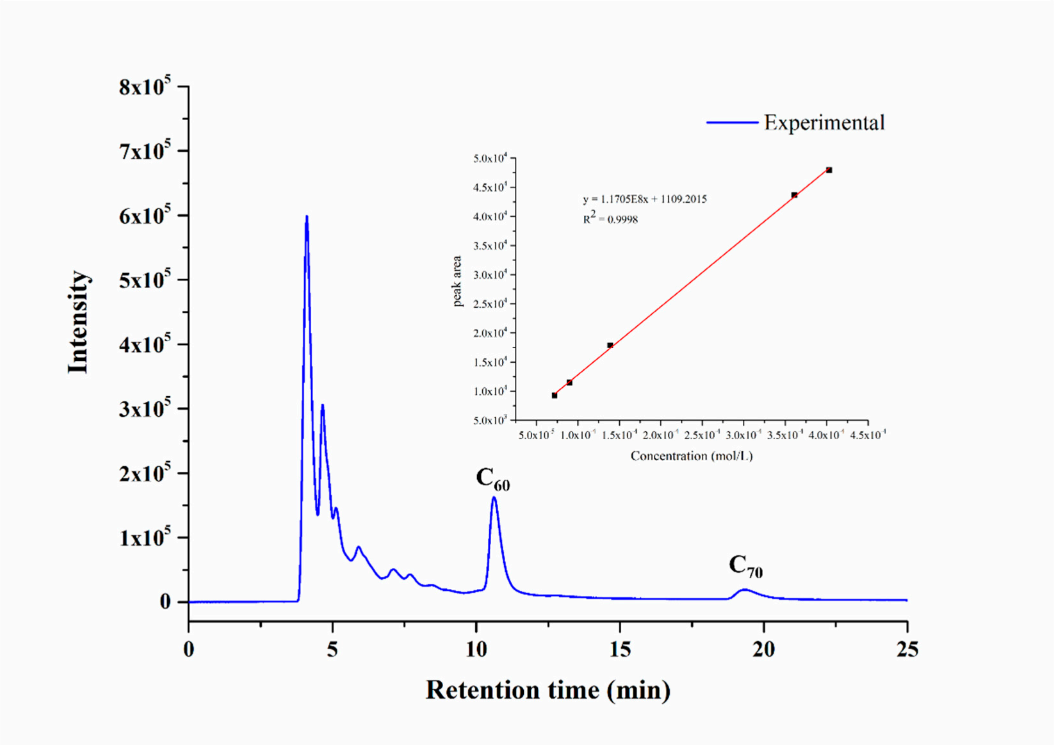

Figure S6. HPLC chromatogram of the soot product from the pyrolysis of $CHCl_3$ at 1530 °C at 330 nm (shown as blue) and the standard curve of fullerene C_{60} inset (shown as red).

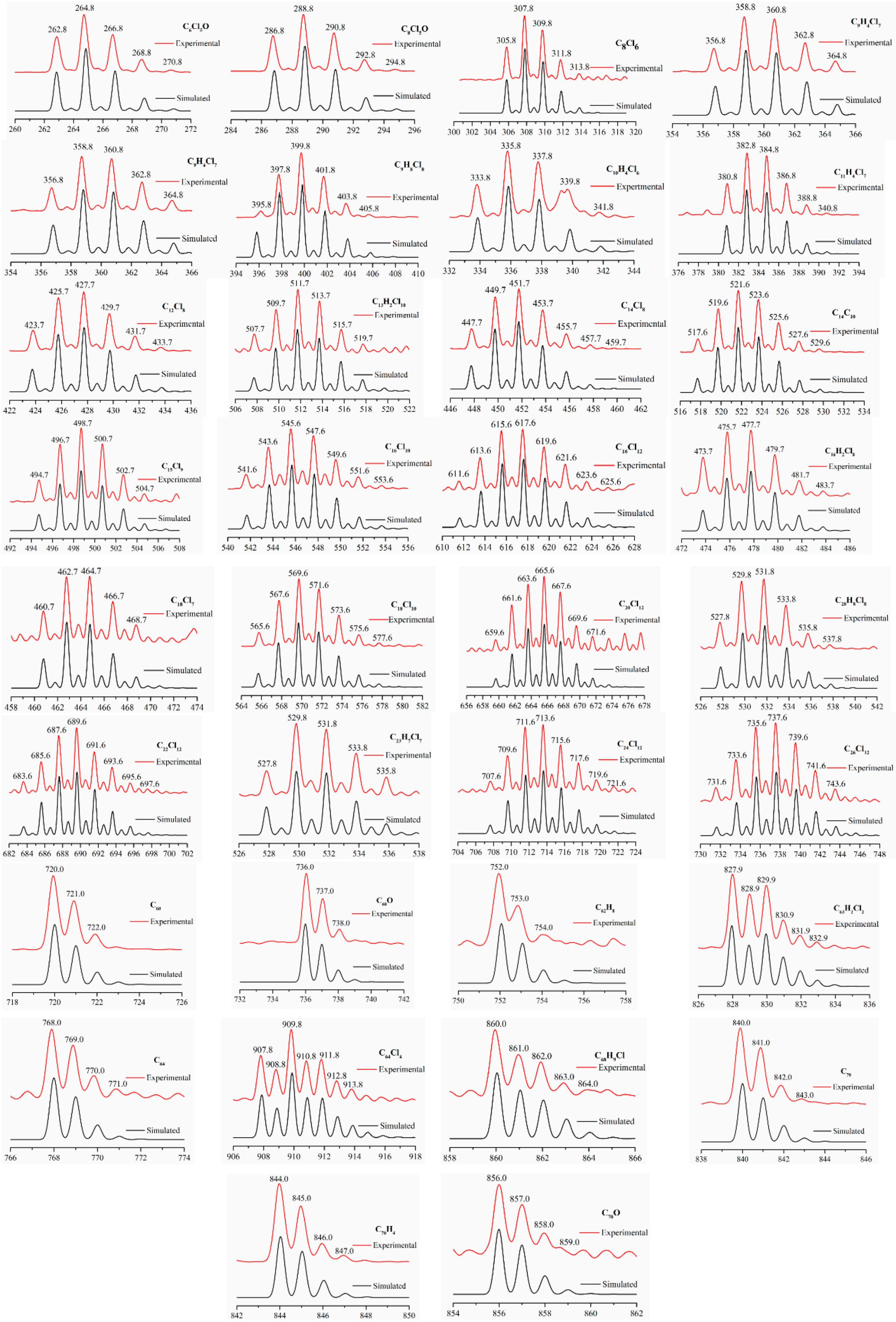

Figure S7. Experimental and theoretically simulated mass spectra of the soot products.

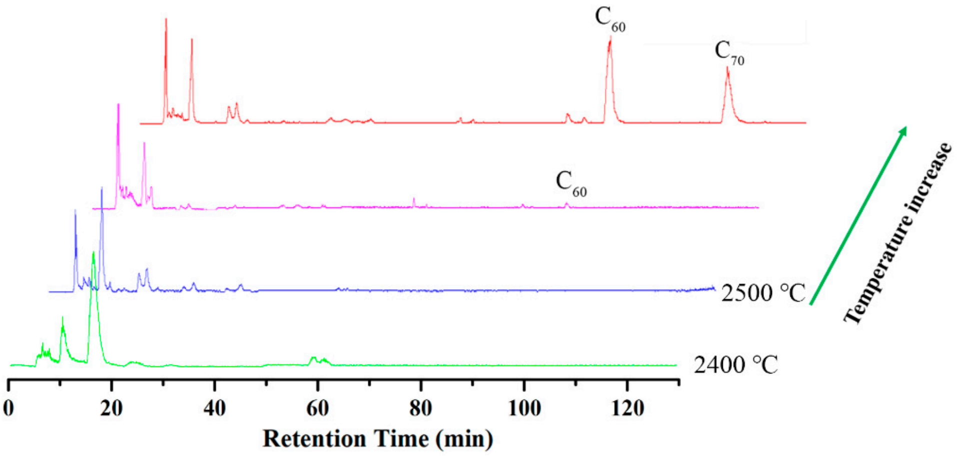

Figure S8. The HPLC-MS chromatograms of the soot products from the pyrolysis of graphite tube at variable pyrolysis temperatures from 2400 °C.
